# Prediction of the Pathogens That Are the Cause of Pneumonia by the Battlefield Hypothesis

**DOI:** 10.1371/journal.pone.0024474

**Published:** 2011-09-01

**Authors:** Takashi Hirama, Takefumi Yamaguchi, Hitoshi Miyazawa, Tomoaki Tanaka, Giichi Hashikita, Etsuko Kishi, Yoshimi Tachi, Shun Takahashi, Keiji Kodama, Hiroshi Egashira, Akemi Yokote, Kunihiko Kobayashi, Makoto Nagata, Toshiaki Ishii, Manabu Nemoto, Masahiko Tanaka, Koichi Fukunaga, Satoshi Morita, Minoru Kanazawa, Koichi Hagiwara

**Affiliations:** 1 Department of Respiratory Medicine, Saitama Medical University, Moroyama, Saitama, Japan; 2 Central Laboratory, Saitama Medical University, Moroyama, Saitama, Japan; 3 Department of General Internal Medicine, Saitama Medical University, Moroyama, Saitama, Japan; 4 Department of Respiratory Medicine, Saitama Medical University International Medical Center, Hidaka, Saitama, Japan; 5 Tsurugashima Ikenodai Hospital, Tsurugashima, Saitama, Japan; 6 Department of Emergency and Acute Medicine, Saitama Medical University International Medical Center, Hidaka, Saitama, Japan; 7 Kan-etsu Hospital, Tsurugashima, Saitama, Japan; 8 Saitama Social Insurance Hospital, Saitama, Saitama, Japan; 9 Department of Biostatistics and Epidemiology, Yokohama City University Medical Center, Yokohama, Kanagawa, Japan; University of Liverpool, United Kingdom

## Abstract

Commensal organisms are frequent causes of pneumonia. However, the detection of these organisms in the airway does not mean that they are the causative pathogens; they may exist merely as colonizers. In up to 50% cases of pneumonia, the causative pathogens remain unidentified, thereby hampering targeting therapies. In speculating on the role of a commensal organism in pneumonia, we devised the battlefield hypothesis. In the “pneumonia battlefield,” the organism-to-human cell number ratio may be an index for the pathogenic role of the organism. Using real-time PCR reactions for sputum samples, we tested whether the hypothesis predicts the results of bacteriological clinical tests for 4 representative commensal organisms: *Streptococcus pneumoniae*, *Haemophilus influenzae*, *Pseudomonas* spp., and *Moraxella catarrhalis*. The cutoff value for the organism-to-human cell number ratio, above which the pathogenic role of the organism was suspected, was set up for each organism using 224 sputum samples. The validity of the cutoff value was then tested in a prospective study that included 153 samples; the samples were classified into 3 groups, and each group contained 93%, 7%, and 0% of the samples from pneumonia, in which the pathogenic role of *Streptococcus pneumoniae* was suggested by the clinical tests. The results for *Haemophilus influenzae*, *Pseudomonas* spp., and *Moraxella catarrhalis* were 100%, 0%, and 0%, respectively. The battlefield hypothesis enabled legitimate interpretation of the PCR results and predicted pneumonia in which the pathogenic role of the organism was suggested by the clinical test. The PCR reactions based on the battlefield hypothesis may help to promote targeted therapies for pneumonia. The prospective observatory study described in the current report had been registered to the University Hospital Medical Information Network (UMIN) registry before its initiation, where the UMIN is a registry approved by the International Committee of Medical Journal Editors (ICMJE). The UMIN registry number was UMIN000001118: A prospective study for the investigation of the validity of cutoff values established for the HIRA-TAN system (April 9, 2008).

## Introduction

Pneumonia is a common disease and is one of the most frequent causes of deaths worldwide. Only 2-dozen species of pathogens are responsible for the most cases of pneumonia. Nevertheless, the causative pathogen cannot be identified by clinical tests such as cultures or serological tests in up to 50% of the cases [1]. Multidisciplinary maneuvers including history taking, physical examination, imaging studies, and epidemiological information complement the clinical test; they help clinicians to list pathogens likely to be the cause and to select antibiotics that can be used to treat them. However, more information than this is often required, especially when a patient is in a serious condition and the pathogens that should be intensively targeted need to be identified without delay.

The polymerase chain reaction (PCR)-based test for purulent sputum is an excellent complementary maneuver. First, if a pathogen is not detected by PCR, the pathogen is less likely to be the causative pathogen, because PCR reaction is sensitive and can detect even a small number of pathogen cells. Second, PCR can detect non-commensal organisms (i.e., organisms that does not colonize in the airway as exemplified by *Mycoplasma pneumoniae* and *Mycobacterium tuberculosis*); if they are detected, they are most likely to be the causative pathogen. Third, PCR is quick, and the result can be delivered to the clinicians in an early phase of the treatment. A major problem associated with a PCR-based test is its inability to discriminate a commensal organism causing pneumonia from the same organism colonizing the airway [2]. These organisms include *Streptococcus pneumoniae* (*S. pneumoniae*), *Haemophilus influenzae* (*H. influenzae*), *Pseudomonas* spp., and *Moraxella catarrhalis* (*M. catarrhalis*) (**Table 1**). Because they are frequent causes of pneumonia, PCR-based tests that can discriminate these 2 states of commensal organisms are desired.

**Table 1:** List of pneumonia-causing pathogens.

	Proportion in community-acquired pneumonia ^[11], [12], [13], [14], [15], [16], [17], [18]^^&^	Proportion in hospital-acquired pneumonia ^[19], [20], [21], [22], [23]^^&^
**Commensal organisms**	**44.94%**	**94.29%**
* Streptococcus pneumoniae*	24.50%	3.40%
* Haemophilus influenzae*	6.04%	5.32%
* Moraxella catarrhalis*	0.62%	2.75%
* Pseudomonas* spp.	0.99%	25.25%
Enterobacteriaceae	3.37%	19.88%
* Klebsiella pneumoniae*		3.74%
others^$^		16.14%
* Stenotrophomonas maltophilia*		2.50%
* Staphylococcus aureus*	2.40%	18.39%
MSSA		4.57%
MRSA		13.82%
Others^#^	7.02%	16.80%
**Non-commensal organisms**	**34.56%**	
* Mycoplasma pneumoniae*	11.18%	
* Chlamydophila pneumoniae*	8.78%	
* Chlamydophila psittaci*	0.36%	
* Legionella* spp.	2.78%	
* Mycobacterium Tuberculosis*	2.64%	
* Pneumocystis jiroveci*	1.12%	
others*	7.70%	
**Unknown or normal flora**	**20.50%**	**5.71%**

& values indicated are the averages from the references cited.

$ includes *Escherichia coli,* *Enterobacter* species, Proteus species, and *Serratia* species.

# includes anaerobes, *Staphylococcus* species (other than *S. aureus*), *Streptococcus* species (other than *S. pneumoniae*), *Acinetobacter* species, and *Aspergillus* species.

*includes viruses and *Coxiella burnetii*.

A definite identification of the causative pathogen of pneumonia is difficult. Instead, a commensal organism is often considered likely to be a causative pathogen when it is (1) detected in the peripheral blood, (2) observed by Gram staining followed by confirmation by sputum culture, (3) phagocytosed by leukocytes in the sputum, or (4) the antigen is detected in the urine antigen test [1], [3], [4], [5], [6], [7], [8], [9], [10]. These criteria have provided legitimate results and thus have been used in clinical practice. Therefore, we considered these criteria as a surrogate endpoint for the identification of the causative pathogen and aimed to establish a PCR-based test of the sputum that provides consistent results. To attain this aim, we devised the “battlefield hypothesis,” in which the ratio of pathogen cells to human inflammatory cells in the sputum is an index for the dominance of the pathogen in the pneumonia “battlefield.” The hypothesis reflects the principle that, in every battle, the relative number of combatants is a major determinant of the current state of battle. In the current study, we demonstrate that the interpretation of the PCR results using the battlefield hypothesis paralleled the results of bacteriological tests. Our results warrant clinical studies that investigate the utility of the PCR-based test in the treatment of pneumonia.

## Methods

### Criteria

Pneumonia: A diagnosis of pneumonia was made when patients presented with both (1) acutely developed symptoms such as fever, cough, sputum, or chest pain and (2) newly developed pulmonary infiltrates in an imaging study [1], [3], [4].

Likely causative pathogen: By reviewing a variety of guidelines [1], [3], [4], [5], [6], [7], [8], [9], we identified 4 criteria that suggested causative pathogens in the bacteriological tests widely used in clinical medicine. A commensal organism that fulfilled at least 1 of the following 4 criteria was considered likely to be a causative pathogen when: (1) an organism was detected in the peripheral blood; (2) a morphologically compatible organism was observed through Gram staining later confirmed by sputum culture; (3) for *S. pneumoniae*, *H. influenzae*, and *M. catarrhalis* only: a morphologically compatible organism coexisted with neutrophils (>100 per visual field at a magnification of 100×) that phagocytosed the organism; or (4) for *S. pneumoniae* only: the urine antigen test was positive.

### Samples

Sputum, induced sputum, or sputum obtained by intra-tracheal aspiration (sputum hereafter) was isolated at the earliest convenience, and at least within 48 h, after the diagnosis of newly developed pneumonia had been established. This excluded the experiment in which a temporal profile of ΔCt_pathogen_ was observed and thus the sputum was serially isolated for several days. Sputum samples were mixed well by pipetting, and divided into 2; one was submitted for a bacteriological test, and the other was submitted for a PCR test. At the same time, a urine sample was submitted for a urine antigen test. Sputum samples that were mostly serous specimens, i.e., with a gross appearance of M1 according to Miller and Jones' classification [10], or with Ct_human_ ≥27 by PCR, were considered inappropriate and were excluded from the study.

### Patients

All patients were (a) 18 years of age or older, (b) diagnosed as having pneumonia by the criteria described above. They also (c) provided sputum that fulfilled the criteria described above, and (d) provided written informed consent. Sample collection was performed from May 2007 to March 2008 (the initial study for determining the cutoff value) and from April 10, 2008 to October 9, 2008 (the prospective study) at the Saitama Medical University Hospital and the participating institutes.

### DNA preparation

The sputum was diluted with an equal volume of phosphate-buffered saline and homogenized by vortexing. A portion of the homogenate (200 µL) was taken and mixed with AL buffer (200 µL; Qiagen, Tokyo, Japan) containing >12 mAU of proteinase K, and the resultant mixture was incubated at 56°C for 2 h. DNA was isolated into 100 µL of TE buffer with the QIAamp DNA Blood Mini Kit (Qiagen).

### Primers and probes

The specificity of the primer and the probe sequences to the 21 target genes was confirmed by a BLAST search of the GenBank database. Each primer and probe set was then tested against a panel that included DNA from 65 respiratory pathogens, 2 drug resistance-related genes, and human genomic DNA (**Table 2**), and was confirmed to exclusively amplify and detect its target DNA.

**Table 2:** List of target genes.

Target	Gene	Oligo	Sequence	Probe conc.	Size	Reference
	(Genbank Accession No.)					
Human	SFTPC (U02948)	F#	5′-GCAGTGCCTACGTCTAAGCTG-3′	0.3 µM	130 bp	
		B	5′-TAGATGTAGTAGAGCGGCACCTC-3′			
		D	FAM-CGAGATGCAGGCTCAGCACCCTC-TAMRA			
Commensal organisms						
*Streptococcus pneumoniae*	Pneumolysin (M17717)	F	5′-CTAAGGCTTGGGACAGAAATGGGC-3′	0.3 µM	172 bp	
		B	5′-CCGCTTACGCACTAGTGGCAAATC-3′			
		D	TET-AGGGAATGTTCGCAATCTCTCTGTCA-BHQ-1			
*Haemophilus influenzae*	16S rRNA (Z22806)	F	5′-TTGACATCCTAAGAAGAGCTCAGAGA-3′	0.3 µM	267 bp	
		B	5′-CTTCCCTCTGTATACGCCATTGTAGC-3′			
		D	TET-ATGGCTGTCGTCAGCTCGTGTT-BHQ-1			
*Moraxella catarrhalis*	copB (U69982)	F	5′-CGTGCGTGTTGACCGTTTTGACTTTA-3′	0.3 µM	298 bp	
		B	5′-CACGCTGCCAAAAATAACTGCCAAAG-3′			
		D	TET-CAGCGGTAACCTAATCTATGCCACTC-BHQ-1			
*Pseudomonas spp.*	16S rRNA (AY486350)	F	5′-GACGGGTGAGTAATGCCTAGGA-3′	0.3 µM	618 bp	[24]
		B	5′-CCACTGGTGTTCCTTCCTATATCT-3′			
		D	TET-AGTGGGGGATCTTCGGACCTCA-BHQ-1			
Non-commensal organisms						
*Mycoplasma pneumoniae*	16S rRNA (NC_000912)	F	5′-AGTAATACTTTAGAGGCGAACGGGTGA-3′	0.3 µM	227 bp	[25]
		B	5′-TCTACTTCTCAGCATAGCTACACGTCA-3′			
		D	FAM-ACCAACTAGCTGATATGGCGCA-TAMRA			
*Chlamydophila pneumoniae*	53KD-antigen (E12535)	F	5′-GCAACCACGGTAGCAACACAAATTA-3′	0.3 µM	364 bp	
		B	5′-AATTGAGCGACGTTTTGTTGCATCT-3′			
		D	FAM-AGCGGCTGTCAAATCTGGAATAAAAG-TAMRA			
*Chlamydophila psittaci*	ompA (X56980)	F	5′-GTATGTTCATGCTTAAGGCTGTTTTCAC-3′	0.1 µM	291 bp	
		B	5′-TCCCACATAGTGCCATCGATTAATAAAC-3′			
		D	TET-CCAGAAGAGCAAATTAGAATAGCGAGCA-BHQ-1			
*Legionella pneumophila*	Mip (S72442)	F	5′-TAACCGAACAGCAAATGAAAGACG-3′	0.1 µM	264 bp	[25]
		B	5′-AAAACGGTACCATCAATCAGACGA-3′			
		D	TET-TGATGGCAAAGCGTACTGCTGAA-BHQ-1			
*Legionella* spp.	16S rRNA (FR799709)	F	5′-AGGCTAATCTTAAAGCGCCAGGCC-3′	0.1 µM	198 bp	[26]
		B	5′-GCATGCTTAACACATGCAAGTCGAAC-3′			
		D	FAM-CATATTCCTACGCGTTACTCACCCGT-TAMRA			
*Mycobacterium tuberculosis*	MPB64 (NC_000962)	F	5′-ATCCGCTGCCAGTCGTCTTCC-3′	0.1 µM	238 bp	[27]
		B	5′-CTCGCGAGTCTAGGCCAGCAT-3′			
		D	TET-CCGGACAACAGGTATCGATAGCGCC-BHQ-1			
*Mycobacterium intracellulare*	ITS 16-23S rRNA (AM709724)	F	5′-AGCACCACGAAAAGCACTCCAATT-3′	0.1 µM	243 bp	
		B	5′-CGAACGCATCAGCCCTAAGGACTA-3′			
		D	FAM-CCTGAGACAACACTCGGTCGATCC-TAMRA			
*Mycobacterium avium*	16S rRNA (M29572)	F	5′-CAAGTCGAACGGAAAGGCCTCT-3′	0.3 µM	257 bp	
		B	5′-GCCGTATCTCAGTCCCAGTGTG-3′			
		D	FAM-TACCGGATAGGACCTCAAGACGC-TAMRA			
*Mycobacterium kansasii*	dnaJ (AB292544)	F	5′-ACCCGTGTGATGAGTGCAAAGGC-3′	0.1 µM	231 bp	
		B	5′-GTAAAGCTGACCGGAACTGTGACG-3′			
		D	TET-AGGACGGACAGCGGATCAGACT-BHQ-1			
*Nocardia* spp.*	16S rRNA (DQ659898)	F	5′-CCTTCGGGTTGTAAACCTCTTTCGAC-3′	0.1 µM	191 bp	
		B	5′-TTGGGGTTGAGCCCCAAGTTTTCA-3′			
		D	FAM-AAGAAGCACCGGCCAACTACGTGC-TAMRA			
*Pneumocystis jiroveci* *	large subunit ribosomal RNA (AF461782)	F	5′-GTGTACGTTGCAAAGTACTCAGAAGA-3′	0.3 µM	346 bp	[28]
		B	5′-GATGGCTGTTTCCAAGCCCA-3′			
		B	5′-GATGGCTGTTTCCAAGCCCA-3′			

*These organisms may be found in a healthy airway on rare occasions. When detected in pneumonic patients, they are very likely to be the causative pathogen. We therefore list them as non-commensal organisms.

# F: forward primer, B: backward primer, D: detection probe. Each primer set was tested against the DNA panel and was confirmed to provide specific amplification only for its target sequence. The panel contains DNA from human, from 65 organisms, and from 2 organisms that harbor drug resistance-related genes; the organisms include *Streptococcus pneumoniae*, *Haemophilus influenzae*, *Moraxella catarrhalis*, *Pseudomonas aeruginosa*, *Klebsiella pneumoniae*, *Stenotrophomonas maltophilia*, *Staphylococcus aureus*, *Mycoplasma pneumoniae*, *Chlamydophila pneumoniae*, *Chlamydophila psittaci*, *Legionella pneumophilia*, *Mycobacterium tuberculosis*, *Mycobacterium intracellulare*, *Mycobacterium avium*, *Mycobacterium kansasii*, *Nocardia asteroids*, *Pneumocystis jiroveci*, *Acinetobacter baumannii*, *Anaerococcus hydrogenalis*, *Aspergillus flavus*, *Aspergillus fumigates*, *Aspergillus niger*, *Bacteroides caccae*, *Bacteroides fragilis*, *Bacteroides thetaiotaomicron*, *Candida albicans*, *Candida parapsilosis*, *Clostridium perfringens*, *Clostridium ramosum*, *Corynebacterium* spp., *Coxiella burnetii*, *Cryptococcus neoformans*, *Eikenella corrodens*, *Enterobacter aerogenes*, *Enterobacter cloacae*, *Enterococcus faecalis*, *Enterococcus faecium*, *Escherichia coli*, *Haemophilus haemolyticus*, *Haemophilus parainfluenzae*, *Klebsiella oxytoca*, *Legionella bozemanii*, *Legionella micdadei*, *Mycobacterium gordonae*, *Neisseria meningitides*, *Peptostreotococcus anaerobius*, *Porphyromonas asaccharolytica*, *Prevotella bivia*, *Prevotella intermedia*, *Proteus mirabilis*, *Serratia marcescens*, *Staphylococcus auricularis*, *Staphylococcus epidermidis*, *Staphylococcus simulans*, *Streptococcus anginosus*, *Streptococcus constellatus*, *Streptococcus intermedius*, *Streptococcus mitis*, *Streptococcus pyogenes*, *Streptococcus uberis*, *Trichosporon asahii*, and *Trichosporon mucoides*; drug resistance-related genes include IMP and mecA.

### Real-time PCR reaction

The PCR reaction solution (25 µL) contained TAKARA Premix Ex Taq (12.5 µL; Takara Bio Inc., Shiga, Japan), primers (300 nM), fluorescence-labeled detection probes (100 nM or 300 nM; **Table 2**), and purified DNA (1 µL). The real-time PCR reactions were performed using the SmartCycler II Real-Time Thermal Cycler (Cepheid, Sunnyvale, CA, USA): for target fragments longer than 500 bp, 95°C for 30 s followed by 40 cycles of 95°C for 10 s, 61°C for 35 s, and 72°C for 25 s; for target fragments shorter than 500 bp, 95°C for 30 s followed by 40 cycles of 95°C for 8 s, 61°C for 25 s, and 72°C for 20 s.

### Statistical analysis

The 95% confidence interval (CI) was calculated using the formula for the binomial probabilities.

### Ethical consideration

The research protocol for the current study was approved by the institutional review boards of the Saitama Medical University Hospital, Saitama International Medical Center, Kan-etsu Hospital, Tsurugashma Ikenodai Hosipital, Ohno Clinic, Kesennuma City Hospital and Saitama Social Insurance Hospital.

All patients were (a) 18 years of age or older, (b) diagnosed as having pneumonia by the criteria described above. They also (c) provided sputum that fulfilled the criteria described above, and (d) provided written informed consent. Sample collection was performed from May 2007 to March 2008 (the initial study for determining the cutoff value) and from April 10, 2008 to October 9, 2008 (the prospective study) at the Saitama Medical University Hospital and the participating institutes.

## Results

### Battlefield hypothesis

**Figure 1:**
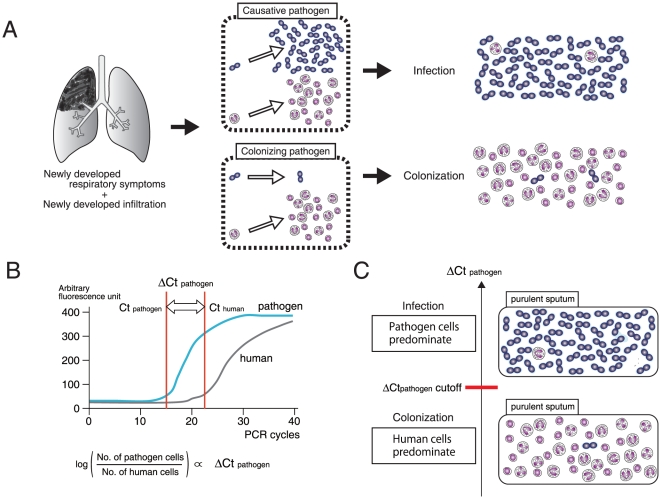
Battlefield hypothesis. (A) When pneumonia occurs, the numbers of both the causative pathogen and human inflammatory cells increase at the inflammation site. Meanwhile, the colonizing pathogen lags behind. The ratio of pathogen to human cells may be a good indicator for the differentiation of the causative pathogen from the colonizing pathogen. (B) The cell number ratio is measurable by quantitative PCR. The Ct (threshold cycle) is the PCR cycle at which a statistically significant fluorescent signal is first observed. Ct_pathogen_ is the Ct for the pathogen-specific gene, Ct_human_ is the Ct for the human-specific gene, and both are log-proportional to the number of the cells (see **Figure 2**). Accordingly, ΔCt_pathogen_ =  −(Ct_pathogen_−Ct_human_) is log-proportional to the ratio of pathogen to human cells. (C) Because ΔCt_pathogen_ indicates the ratio of pathogen to human cells, we may be able to determine the ΔCt_pathogen_ cutoff, a ΔCt_pathogen_ value above which a pathogenic role of the pathogen in pneumonia is strongly suggested.

The battlefield hypothesis assumes that the ratio of the cells of a commensal organism to human inflammatory cells is an index of the pathogenic role of the organism (**Figure 1A**). In a quantitative PCR-based system, with the use of primers and probes that specifically quantitate the cell numbers of each pathogen or human, the ratio of pathogen cells to human cells is log-proportional to ΔCt_pathogen_ = − (Ct_pathogen_−Ct_human_) (**Figure 1B**). As predicted by the battlefield hypothesis, the ΔCt_pathogen_ cutoff is a threshold value that can be used to discriminate a commensal organism with a pathogenic role from the same organism simply colonizing the airway (**Figure 1C**).

### Determination of the ΔCt_pathogen_ cutoff

**Figure 2:**
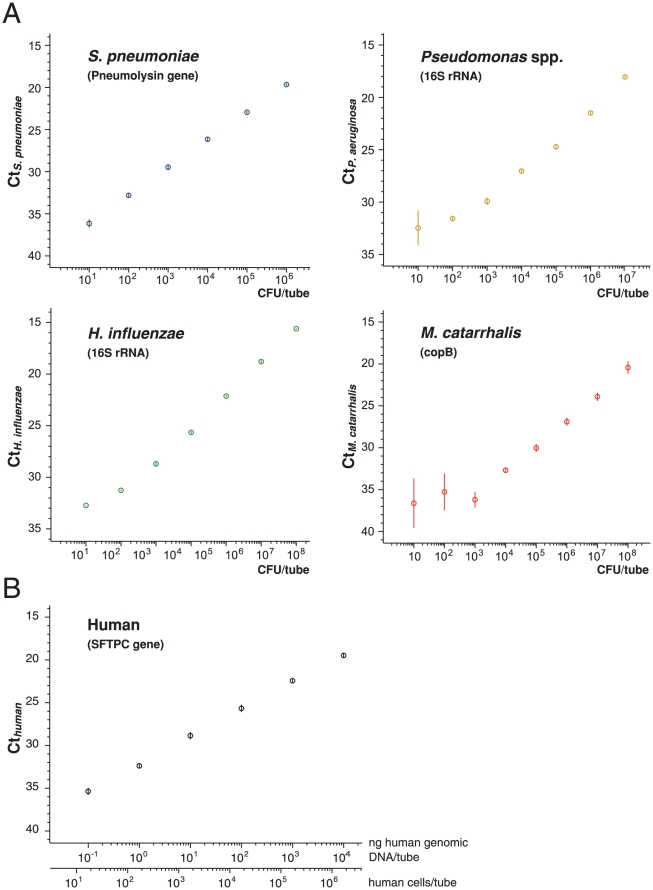
Relationship between cell number and Ct. Log-linear relationships between the copy number of pathogen-specific sequence and Ct_pathogen_ and between the copy number of the human-specific sequence and Ct_human_. (A) *S. pneumoniae*, *H. influenzae*, *Pseudomonas* spp., or *M. catarrhalis* was suspended in sputum. DNA was then purified from the suspension, and the target sequences specific to each organism were amplified by PCR. A log-linear relationship indicates that the sputum does not contain molecules that inhibit isolation of DNA or exponential amplification by PCR. Experiments were done in triplicate. A bar indicates standard deviation. (B) Human genomic DNA isolated from sputum was serially diluted, and a DNA sequence in the human SFTPC gene (arbitrarily selected from human genes, of which sequence is specific to human by BLAST search of GenBank database; **Table 2**) was amplified. A log-linear relationship indicates that the sputum does not contain molecules that inhibit isolation of DNA or exponential amplification by PCR.

We confirmed that the primers and probes used in the current study were specific to 4 representative commensal organisms (*S. pneumoniae*, *H. influenzae*, *Pseudomonas* spp., and *M. catarrhalis*) and to human (**Table 2**), and were able to quantify their cell numbers in sputum (**Figure 2**).

**Figure 3:**
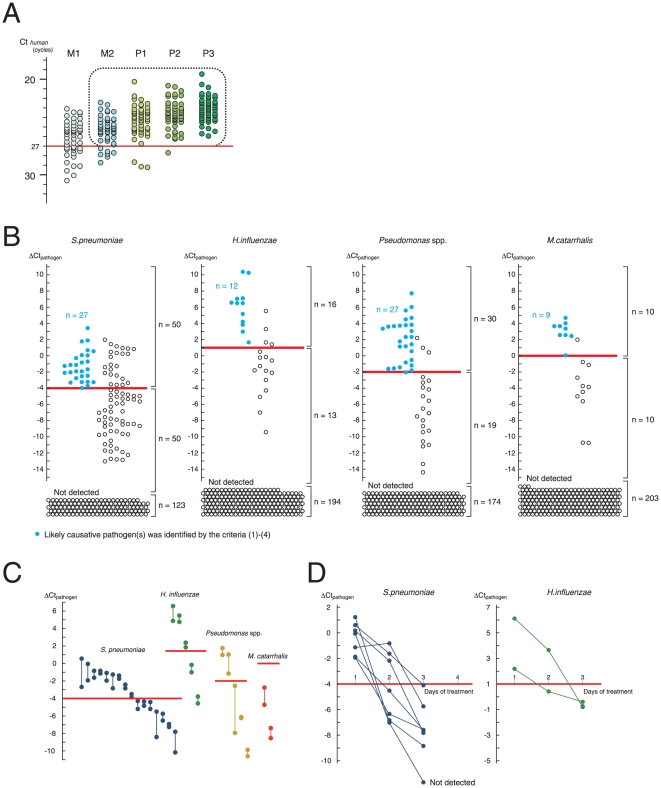
Determination of the ΔCt cutoff. (A) Selection of purulent sputum. Sputum was classified by its gross appearance, with 50 samples studied for each classification. Purulent sputum had a Ct_human_ <27 (>7×10^3^ human cells/µL of sputum; **Figure 2B**). Samples with M2–P3 appearance as well as a Ct_human_ <27 (enclosed by a dotted line) were studied further. Classification of the gross appearance of the sputum (M1, M2, P1, P2, and P3) are according to Miller and Jones [10]. (B) Determination of the ΔCt cutoff. ΔCt_pathogen_ was measured for 4 representative commensal organisms (n = 223). Samples from patients with pneumonia in which a likely causative pathogen was identified using criteria (1)–(4) (see Methods) are shown as blue circles, and samples from patients with pneumonia in which none of criteria (1) – (4) was fulfilled were shown as white circles. The ΔCt_pathogen_ cutoff (a red line) was defined as the smallest ΔCt_pathogen_ for the blue circles. Sputum in which the pathogen was not detected and thus ΔCt_pathogen_ was not assigned is shown at the bottom (labeled as “Not detected”). (C) Reproducibility of ΔCt_pathogen_ measurements. Duplicate samples were isolated from a single patient in a single day (n = 28), and each of the duplicate samples was independently measured for ΔCt_pathogen_. Both of the measurements provided ΔCt_pathogen_ located on the same side (above or below) of the cutoff. Red line: the cutoff for each organism. (D) Temporal profile of ΔCt_pathogen_ during antibiotic treatment. A single sample set contains multiple sputum samples isolated from a single patient during antibiotic treatment. A total of 9 consecutive sample sets that included 7 of pneumonia with ΔCt_pathogen_ for *S. pneumoniae* > cutoff and 2 of pneumonia with ΔCt_pathogen_ for *H. influenzae* > cutoff at day 1 were studied. ΔCt_pathogen_ decreased to below the cutoff in the course of treatment.

We then determined a PCR criterion that can select purulent sputum suitable for the test, and exclude M1 samples that are mostly saliva. We found that P1–P3 samples almost entirely had a Ct_human_ of <27 (**Figure 3A**). In the current study, sputum samples that were classified as M2–P3 by the naked eye and had a Ct_human_ of <27 were considered suitable and investigated further.

We then screened pathogens using PCR (**Figure 3B** **)**. Blue circles represent the samples isolated from patients in whom the organism was identified as a likely causative pathogen on the basis of the said criteria (1) – (4). By setting the cutoff as the smallest value of ΔCt_pathogen_, the samples were classified into 3 groups: (i) Samples with ΔCt_pathogen_ ≥ cutoff, in which case the organism was considered to have a high chance of being a causative pathogen; (ii) Samples with ΔCt_pathogen_ < cutoff, in which case the organism had a small chance of being a causative pathogen; (iii) samples in which the organism was not detected, in which case the pathogen was unlikely to be a causative pathogen.

The procedure for preparing DNA from the sputum included homogenization of the sputum by digestion of its proteins, so that the procedure was less affected by the viscosity of the sputum. However, patients can expectorate sputum of different qualities at different times. To study the variation between the samples, we studied consecutive sample pairs, in which a pair contains 2 samples expectorated in a single day, and graphed 28 pairs in which 1 of 4 commensal organisms was detected. For each case examined, both samples in a pair were observed to be located on the same side (either above or below) of the cutoff (**Figure 3C**). This suggested that the variation between the samples did not affect to a large extent to the classification of the sputum into 3 groups.

According to the battlefield hypothesis, the ratio of pathogen to human cells is predicted to decrease with the clearance of bacterial cells by inflammatory cells. We thus investigated the change in ΔCt_pathogen_ in the early days of treatment by studying 9 consecutive patients (**Figure 3D**). In all of them, the ΔCt_pathogen_ decreased during the treatment. This was consistent with the speculation described above.

### Prospective study

**Figure 4:**
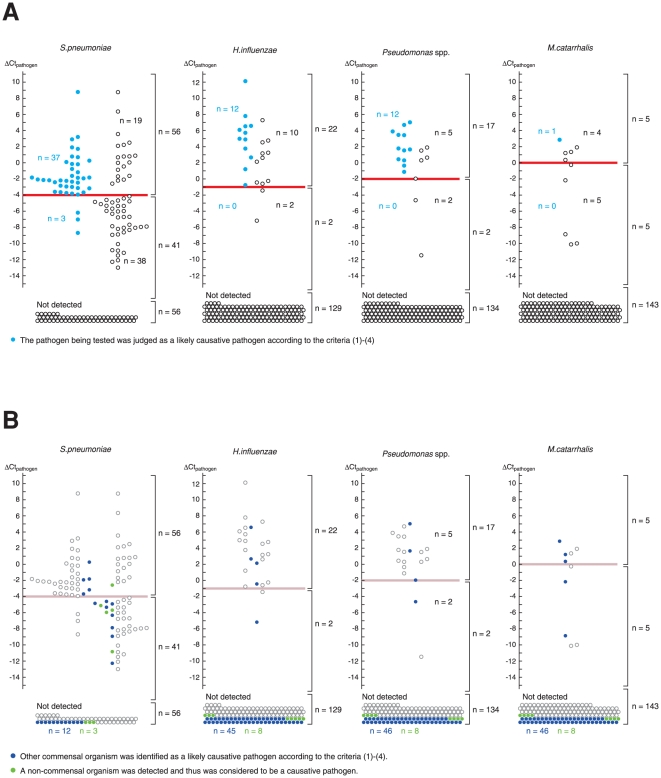
A prospective study. (A) ΔCt_pathogen_ for each commensal organism (n = 153). Samples in which real-time PCR failed to detect the organism are shown at the bottom (“Not detected”). The ΔCt_pathogen_ cutoff demarcated well the samples obtained from the patients in whom the likely causative pathogen was identified by criteria (1) – (4). (B) Interrelationship between the pathogens detected. Samples obtained from the patients in whom the other 3 commensal organisms were identified as a likely causative pathogen or samples in which a non-commensal organism was detected by real-time PCR are colored. Most of the colored circles are located below the cutoff line.

In order to confirm whether the suggested cutoff values reproducibly identified pneumonia for cases in which the organism had a good chance of being a likely causative pathogen, we designed a prospective observatory study (see **Table 3** for the protocol). Here, we expanded the PCR reactions to cover 11 non-commensal organisms and named the system HIRA-TAN (HIRA = human cell-controlled identification of the respiratory agent; “TAN” means sputum in Japanese; **Table 2**). A total of 153 samples were enrolled (**Table 4**). The primary endpoint was the percentage of sputum that had a ΔCt_pathogen_ value greater than the cutoff value, when *S. pneumoniae* was the likely causative pathogen (on the graph, it is the percentage of blue circles above the red line). The proportion was 0.93 (37/40; 95% CI, 0.80–0.97) (**Figure 4A**). The secondary endpoints were the percentages for the other 3 commensal organisms. They were 1.0 (12/12; 95% CI, 0.74–0.998) for *H. influenzae*, 1.0 (12/12; 95% CI 0.74–0.998) for *Pseudomonas* spp., and 1.0 (1/1; 95% CI undetermined) for *M. catarrhalis*. These results indicated that the test was strong at predicting the likelihood of the organism being a likely causative pathogen; when ΔCt_pathogen_ was greater than the cutoff value, *S. pneumoniae* had a 66% chance (37 blue circles/56 blue + white circles) of being a likely causative pathogen; when ΔCt_pathogen_ was smaller than the cutoff value, the chance was 7% (3/41); when *S. pneumoniae* was not detected by PCR, the chance was 0% (0/56) (**Figure 4A**). Comparative results were obtained for all of the other organisms.

**Table 3:** Protocol for the prospective observatory study.

Study design	A multi-institutional study. Sputum was collected from all outpatients and inpatients (≥18 years of age) who developed pneumonia.
Study period and sample size^#^	Between April 10, 2008 and April 9, 2009. The study will be terminated before April 9, 2009, when the total number of patients reaches 300, or when the number of patients with pneumonia in which *S. pneumoniae* is the likely causative pathogen reaches 40.
Eligibility criteria	All patients who provided sputum classified as M2, P1, P2 or P3 by the criteria of Miller and Jones [10]. Sputum is isolated at the earliest convenience, at least within 48 h, after the diagnosis of newly developed pneumonia is established. Sputum is divided into 2 samples; one is submitted for a bacteriological test including Gram staining and culture, and the other is submitted for the HIRA-TAN test. The samples are also submitted for urine antigen test for *S. pneumoniae*.
Primary endpoint	The proportion of samples in which *S. pneumoniae* was detected with a ΔCt_pathogen_ ≥ cutoff to those in which *S. pneumoniae* was shown to be a likely causative pathogen.
Secondary endpoints	The proportion of the samples in which *H. influenzae*, *M. catarrhalis*, or *Pseudomonas* spp. was detected with a ΔCt_pathogen_ ≥ cutoff to those in which each was shown to be a likely causative pathogen.

# The number was determined so that the primary endpoint is evaluated at a resolution of 0.1.

**Table 4:** Patients' characteristics.

Age (y)	(Mean [min-max])	65.9 (18–96)
Sex	Male (%)	83 (54.2%)
	Female (%)	70 (45.8%)
Sputum (Miller and Jones' classification)		
	M2 (%)	31 (20.3%)
	P1 (%)	38 (24.8%)
	P2 (%)	22 (14.4%)
	P3 (%)	62 (40.5%)
Ct_human_ (mean [min-max])		24.58 (20.58–26.85)
Type of pneumonia		
	Community-acquired pneumonia (CAP) (%)	113 (73.9%)
	Healthcare-associated pneumonia (HCAP) (%)	24 (15.7%)
	Hospital-associated pneumonia (HAP) (%)	16 (10.5%)
Serum CRP (mg/dL) (Mean [min-max])		11.0 (0.1–45.39)
Radiological feature	Monolobar infiltrate	71 (46.4%)
	Multilobar infiltrates	82 (53.6%)
Underlying disorder	Neoplastic disease	5 (3.3%)
	Congestive heart failure	18 (11.8%)
	Cerebrovascular disease	25 (16.3%)
	Renal disease	9 (5.9%)
	Liver disease	4 (2.6%)
	Chronic respiratory disease	49 (26.1%)
	Lung cancer	9 (5.9%)
	Collagen vascular disease	19 (12.4%)
	Steroid usage	13 (8.5%)
	Diabetes	15 (9.8%)

It is interesting to study the range of ΔCt_pathogen_ values observed when other organisms were a likely causative pathogen. In most of the cases, ΔCt_pathogen_ was less than the cutoff or the organism was not detected (**Figure 4B**). This is consistent with our knowledge that a single pathogen plays a dominant pathogenic role in most cases of pneumonia.

## Discussion

If PCR can provide legitimate information on a detected commensal organism (i.e., pathogenic or colonizing), it will become a more important test in clinical practice. The interpretation of PCR results on the basis of the battlefield hypothesis provided such information, which can be summarized as follows: (i) an organism that presented a ΔCt_pathogen_ value above the cutoff had a good chance of being the likely causative pathogen, (ii) an organism that presented a ΔCt_pathogen_ below the cutoff had a small chance of being the likely causative pathogen, and (iii) an organism that was not detected was very unlikely to be the likely causative pathogen.

In the current study, we studied 4 commensal organisms and found that the battlefield hypothesis worked well for all of them. However, it will be important to determine whether the hypothesis is applicable to other commensal organisms, including *Staphylococcus aureus* and *Klebsiella pneumoniae*, which are frequent causes of pneumonia. We have expanded the list of target pathogens for the HIRA-TAN to include these commensal organisms, and the studies are in progress. Although the results are preliminary, the battlefield hypothesis seems to be working for these 2 organisms as well. Further studies are however necessary to confirm the results.

The immune reaction may be weak in the elderly and in immunocompromised hosts; therefore, the ΔCt_pathogen_ cutoff suitable for these people may be different from that for other individuals. In patients with weak immune reactions, the increase in the number of inflammatory cells may be small. Hence, the value of Ct_human_ may increase, thereby resulting in a larger ΔCt_pathogen_ according to the equation ΔCt_pathogen_ = −(Ct_pathogen_−Ct*_human_*). Therefore, the use of the ΔCt_pathogen_ cutoff determined for patients with normal immunity is considered to provide a margin of safety for those with compromised immunity, which will be desirable in clinical practice.

The likely causative pathogen was not identified for half of the samples with a ΔCt_pathogen_ above the cutoff (white circles above the red line, **Figure 2A**). Some of these samples are located in the topmost part of the graph, and have a large ratio of pathogen-to-human cell numbers. One possibility is that criteria (1) – (4) may have a large false negative rate and failed to identify a portion of the pathogens causing pneumonia [1]. Studies using sputum isolated by bronchoscopy will be necessary to investigate this issue.

An important question is whether the information provided by the battlefield hypothesis and its implementation as HIRA-TAN will be useful in clinical practice. We found that HIRA-TAN was readily applicable to clinical practice; the consumables are 2 US dollars/pathogen and the results have been delivered to clinicians in 4 h at our institute. Considering that HIRA-TAN covers pathogens frequently involved in pneumonia, and that the results can be delivered early in the course of treatment, we anticipate that patients with pneumonia will benefit from HIRA-TAN, thus warranting clinical studies.

In conclusion, the battlefield hypothesis enabled legitimate interpretation of PCR results and predicted pneumonia in which the organism is a likely causative pathogen. By providing information on the pathogens in pneumonia, HIRA-TAN will help promoting targeted therapies for pneumonia.

## References

[1] File TM (2003). Community-acquired pneumonia.. Lancet.

[2] Seifert HS, DiRita VJ (2006). Evolution of Microbial Pathogens: Blackwell Publishing.

[3] American Thoracic Society; Infectious Diseases Society of America (2005). Guidelines for the management of adults with hospital-acquired, ventilator-associated, and healthcare-associated pneumonia.. Am J Respir Crit Care Med.

[4] Mandell LA, Marrie TJ, Grossman RF, Chow AW, Hyland RH (2000). Canadian guidelines for the initial management of community-acquired pneumonia: an evidence-based update by the Canadian Infectious Diseases Society and the Canadian Thoracic Society. The Canadian Community-Acquired Pneumonia Working Group.. Clin Infect Dis.

[5] Woodhead M, Blasi F, Ewig S, Huchon G, Ieven M (2005). Guidelines for the management of adult lower respiratory tract infections.. Eur Respir J.

[6] Masterton RG, Galloway A, French G, Street M, Armstrong J (2008). Guidelines for the management of hospital-acquired pneumonia in the UK: report of the working party on hospital-acquired pneumonia of the British Society for Antimicrobial Chemotherapy.. J Antimicrob Chemother.

[7] Miyashita N, Matsushima T, Oka M, Japanese Respiratory S (2006). The JRS guidelines for the management of community-acquired pneumonia in adults: an update and new recommendations.. Intern Med.

[8] Watanabe A, Yanagihara K, Kohno S, Matsushima T (2008). Multicenter survey on hospital-acquired pneumonia and the clinical efficacy of first-line antibiotics in Japan.. Intern Med.

[9] Calandra T, Cohen J (2005). The international sepsis forum consensus conference on definitions of infection in the intensive care unit.. Crit Care Med.

[10] Miller DL, Jones R (1963). A study of techniques for the examination of sputum in a field survey of chronic bronchitis.. Am Rev Respir Dis.

[11] French Society of Infectious Diseases (2001). What should the initial antibiotherapy for acute community-acquired pneumonia be? How should it be reassessed in case of failure, given the evolution of responsible pathogens and the resistance of pneumococci? Should combined treatment be used?. Med Mal Infect.

[12] Park DR, Sherbin VL, Goodman MS, Pacifico AD, Rubenfeld GD (2001). The etiology of community-acquired pneumonia at an urban public hospital: influence of human immunodeficiency virus infection and initial severity of illness.. J Infect Dis.

[13] Miyashita N, Fukano H, Niki Y, Matsushima T, Okimoto N (2001). Etiology of community-acquired pneumonia requiring hospitalization in Japan.. Chest.

[14] Luna CM, Famiglietti A, Absi R, Videla AJ, Nogueira FJ (2000). Community-acquired pneumonia: etiology, epidemiology, and outcome at a teaching hospital in Argentina.. Chest.

[15] Wattanathum A, Chaoprasong C, Nunthapisud P, Chantaratchada S, Limpairojn N (2003). Community-acquired pneumonia in southeast Asia: the microbial differences between ambulatory and hospitalized patients.. Chest.

[16] Ruiz-Gonzalez A, Falguera M, Nogues A, Rubio-Caballero M (1999). Is Streptococcus pneumoniae the leading cause of pneumonia of unknown etiology? A microbiologic study of lung aspirates in consecutive patients with community-acquired pneumonia.. Am J Med.

[17] Lim WS, Macfarlane JT, Boswell TC, Harrison TG, Rose D (2001). Study of community acquired pneumonia aetiology (SCAPA) in adults admitted to hospital: implications for management guidelines.. Thorax.

[18] Scott JA, Hall AJ, Muyodi C, Lowe B, Ross M (2000). Aetiology, outcome, and risk factors for mortality among adults with acute pneumonia in Kenya.. Lancet.

[19] Soo Hoo GW, Wen YE, Nguyen TV, Goetz MB (2005). Impact of clinical guidelines in the management of severe hospital-acquired pneumonia.. Chest.

[20] Leroy O, Jaffre S, D'Escrivan T, Devos P, Georges H (2003). Hospital-acquired pneumonia: risk factors for antimicrobial-resistant causative pathogens in critically ill patients.. Chest.

[21] Watanabe A, Fujimura S, Kikuchi T, Gomi K, Fuse K (2007). Evaluation of dosing designs of carbapenems for severe respiratory infection using Monte Carlo simulation.. J Infect Chemother.

[22] Ioanas M, Cavalcanti M, Ferrer M, Valencia M, Agusti C (2003). Hospital-acquired pneumonia: coverage and treatment adequacy of current guidelines.. Eur Respir J.

[23] Herer B, Fuhrman C, Demontrond D, Gazevic Z, Housset B (2001). Diagnosis of nosocomial pneumonia in medical ward: repeatability of the protected specimen brush.. Eur Respir J.

[24] Spilker T, Coenye T, Vandamme P, LiPuma JJ (2004). PCR-based assay for differentiation of Pseudomonas aeruginosa from other Pseudomonas species recovered from cystic fibrosis patients.. J Clin Microbiol.

[25] Morozumi M, Nakayama E, Iwata S, Aoki Y, Hasegawa K (2006). Simultaneous detection of pathogens in clinical samples from patients with community-acquired pneumonia by real-time PCR with pathogen-specific molecular beacon probes.. J Clin Microbiol.

[26] Templeton KE, Scheltinga SA, Beersma MF, Kroes AC, Claas EC (2004). Rapid and sensitive method using multiplex real-time PCR for diagnosis of infections by influenza a and influenza B viruses, respiratory syncytial virus, and parainfluenza viruses 1, 2, 3, and 4.. J Clin Microbiol.

[27] Takahashi T, Nakayama T (2006). Novel technique of quantitative nested real-time PCR assay for Mycobacterium tuberculosis DNA.. J Clin Microbiol.

[28] Wakefield AE, Pixley FJ, Banerji S, Sinclair K, Miller RF (1990). Detection of Pneumocystis carinii with DNA amplification.. Lancet.

